# Assessing the Efficacy and Safety of a Digital Therapeutic for Symptoms of Depression in Adolescents: Protocol for a Randomized Controlled Trial

**DOI:** 10.2196/48740

**Published:** 2023-11-16

**Authors:** Daniella J Furman, Shana A Hall, Claudia Avina, Vera N Kulikov, Jessica I Lake, Aarthi Padmanabhan

**Affiliations:** 1 Limbix Health, Inc San Francisco, CA United States; 2 Big Health, Inc San Francisco, CA United States

**Keywords:** randomized controlled trial, depression, adolescent, youth, mental health, digital therapeutic, behavioral activation, cognitive behavioral therapy, virtual trial, efficacy, treatment, digital health, intervention, mental illness

## Abstract

**Background:**

Depression is a serious, prevalent, recurrent, and undertreated disorder in adolescents. Low levels of treatment seeking and treatment adherence in this age group, combined with a growing national crisis in access to mental health care, have increased efforts to identify effective treatment alternatives for this demographic. Digital health interventions for mental illness can provide cost-effective, engaging, and accessible means of delivering psychotherapy to adolescents.

**Objective:**

This protocol describes a virtual randomized controlled trial designed to evaluate the efficacy and safety of a self-guided, mobile app–based implementation of behavioral activation therapy, SparkRx, for the adjunct treatment of symptoms of depression in adolescents.

**Methods:**

Participants are recruited directly through web-based and print advertisements. Following eligibility screening and consenting, participants are randomly assigned to a treatment arm (SparkRx) or a control arm (assessment-enhanced usual care) for 5 weeks. The primary efficacy outcome, total score on the 8-item Patient Health Questionnaire (PHQ-8), is assessed at the end of the 5-week intervention period. Additional participant-reported outcomes are assessed at baseline, the postintervention time point, and 1-month follow-up. The safety of the intervention is assessed by participant report (and legal guardian report, if the participant is younger than 18 years) and by patterns of symptom deterioration on the PHQ-8, as part of a larger clinical safety monitoring protocol. The primary efficacy outcome, total PHQ-8 score at the postintervention time point, will be compared between SparkRx and enhanced usual care arms using mixed effect modeling, with baseline PHQ-8 and current antidepressant medication status included as covariates. Secondary efficacy outcomes, including the proportion of participants exhibiting treatment response, remission, and minimal clinically significant improvement (all derived from total PHQ-8 scores), will be compared between groups using chi-square tests. Symptom severity at 1-month follow-up will also be compared between arms. Planned subgroup analyses will examine the robustness of treatment effects to differences in baseline symptom severity (PHQ-8 score <15 or ≥ 15) and age (younger than 18 years and older than 18 years). The primary safety outcome, the number of psychiatric serious adverse events, will be compared between trial arms using the Fisher exact test. All other adverse events will be presented descriptively.

**Results:**

As of May 2023, enrollment into the study has concluded; 223 participants were randomized. The analysis of the efficacy and safety data is expected to be completed by Fall 2023.

**Conclusions:**

We hypothesize that the results of this trial will support the efficacy and safety of SparkRx in attenuating symptoms of depression in adolescents. Positive results would more broadly support the prospect of using accessible, scientifically validated, digital therapeutics in the adjunct treatment of mental health disorders in this age range.

**Trial Registration:**

ClinicalTrials.gov NCT05462652; https://clinicaltrials.gov/study/NCT05462652

**International Registered Report Identifier (IRRID):**

DERR1-10.2196/48740

## Introduction

Depression is among the most prevalent mental health disorders in adolescents and young adults [1,2], with global rates rising sharply in the past decade [3]. Symptoms of the disorder can contribute in significant ways to the disruption of social and familial relationships, decreased quality of life and physical health, poor academic and occupational outcomes, and increased risk of substance abuse and self-harm [4-11]. Alarmingly, suicide is now among the leading causes of death in this age group [12-14], highlighting a critical need for the development and deployment of effective and accessible treatments to affected individuals.

Despite the high rate of depression among adolescents, levels of treatment seeking and adherence are low for both psychotherapy and pharmaceutical treatment [15-17], likely due in part to concerns over privacy or stigma [18,19] and side effects [20,21], respectively. Simultaneously, access to effective mental health care is often limited [22-24], with a documented shortage of mental health providers [25-27], long waitlists [28-33], and prohibitive treatment costs [34,35]. For young patients, logistical barriers may also exist for seeking or accessing traditional forms of care, such as limited caregiver time, and conflicting responsibilities, such as school and other activities. An evidence-based self-guided psychotherapy program delivered via personal mobile device may be a promising and scalable option as an adjunct to the standard of care; such a program, if effective, would be expected to overcome many of the existing limitations in access to mental health care, adherence, and safety. As with other digital health interventions, such a program could be less stigmatizing, leading to higher rates of engagement and self-disclosure [36-39].

Cognitive behavioral therapy (CBT), a recommended form of treatment for adolescent depression by the American Academy of Pediatrics, has been shown to be effective when implemented in a digital format [40-42]. Behavioral activation, a key component of CBT, may be particularly well suited for deployment as a digital therapeutic, as it is fundamentally individualized, self-driven, and self-monitored. Specifically, behavioral activation emphasizes engagement in adaptive and personally meaningful activities to increase feelings of reward, mastery, or goal advancement; additional components focus on techniques for reducing harmful or avoidance behaviors commonly associated with depression [43-46]. Importantly, evidence supports behavioral activation therapy on its own as efficacious in the treatment of depression [47]. SparkRx (Limbix Health, Inc), an investigational mobile app–based digital therapeutic, was developed to deliver behavioral activation programming and psychoeducation to adolescents with symptoms of depression as an adjunct to usual care.

This randomized controlled trial (RCT) is designed to test the efficacy and safety of SparkRx. To test the efficacy of the intervention, we will compare self-reported symptom severity (total score 8-item Patient Health Questionnaire [PHQ-8] [48]) between adolescents who receive SparkRx and adolescents who continue their usual care for symptoms of depression. We hypothesize that the SparkRx group will exhibit (1) lower PHQ-8 scores at the postintervention time point, (2) higher rates of treatment response defined as a 10-point decrease in PHQ-8 score from baseline, (3) higher rates of remission defined as a postintervention PHQ-8 score less than 5, (4) lower PHQ-8 scores at 1-month follow-up, and (5) higher rates of clinically meaningful reduction in symptom severity, defined as a 5-point or greater reduction in PHQ-8 score from baseline to postintervention. We also hypothesize that SparkRx users will, on average, demonstrate a minimal clinically important difference at the postintervention time point, defined as a 5-point reduction from baseline. To test the safety of SparkRx, the number of serious adverse events (SAEs) reported during the intervention period will be compared between treatment groups. We hypothesize that participants who receive SparkRx will report no more of these events than those who continue their usual care for symptoms of depression.

## Methods

### Study Overview

This parallel-group, superiority, and single-blind (investigator) RCT compares the efficacy and safety of an investigational digital therapeutic (SparkRx) with assessment-enhanced usual care (eUC) for symptoms of depression in adolescents (13-21 years). Participants are randomly assigned to receive 5 weeks of access to either SparkRx or a control app with no therapeutic content in addition to continuing their usual care for symptoms of depression. Participant-reported efficacy outcomes are obtained at baseline, weekly during the intervention period, at the postintervention time point, and at 1-month follow-up. Safety is monitored throughout study participation using a combination of explicit assessment of symptom deterioration and side effects and triage of other incoming information for potential clinical concerns. The RCT is conducted entirely virtually. Study procedures were preregistered on ClinicalTrials.gov (NCT05462652). The study is overseen by an independent Data and Safety Monitoring Board.

### Ethics Approval

Study procedures were approved by the Advarra institutional review board prior to participant enrollment (Pro00061682).

### Sample Size

G*Power [49] was used to calculate the estimated sample size based on the primary outcome—total PHQ-8 score at postintervention. Using a 2-sample *t* test approach and assuming a 2-tailed α set at .05, a total of 200 participants (100 in each study arm) was estimated to provide 80% power to detect a moderate effect size of at least *d*=0.4. Given an anticipated 10% rate of attrition, the target sample size was determined to be 220 (110 randomized to each trial arm).

### Participants

The inclusion criteria are assessed via self-report and legal guardian report (if applicable, ie, participant is under the age of 18 years) and include (1) aged 13-21 years; (2) PHQ-8 score ≥5 at eligibility screening; (3) English fluency and literacy; (4) access to a compatible smartphone (or other device) and operating system (ie, capable of installing the app from the Google Play or Apple App Store) and regular internet access (most app content is usable offline, but internet access is required to submit weekly check-in PHQ-8 and safety data); (5) willing and able to provide informed consent or assent and, if required, have a legal guardian willing and able to provide informed consent; (6) under the care of a US-based licensed health care provider and willing and able to provide contact information for the provider and sign a Health Insurance Portability and Accountability Act (HIPAA) release that allows Limbix to contact provider; (7) willing and able to provide the information required for study enrollment (eg, all responses to initial PHQ-8 assessment and current antidepressant medication status); and (8) located in the continental United States, Hawaii, or Alaska and not planning to leave the United States during the study period (through 1-month follow-up assessments, up to 11 weeks after eligibility screening).

The exclusion criteria are assessed via self-report and legal guardian report (if applicable) and include (1) a diagnosis of (or treatment for) bipolar disorder, posttraumatic stress disorder, psychotic disorder, substance use disorder, or eating disorder within the 12 months prior to eligibility screening; (2) recent change in psychotropic medication (past 30 days) or plans to change psychotropic medication or other treatment for a mental health disorder during the 5-week intervention period; (3) recent participation (past 60 days) in or plans to initiate participation in any other mental health intervention research during the intervention period; (4) suicide attempt within the past year; (5) active suicidal ideation with intent; (6) having a sibling in the study; (7) previous participation in user testing or clinical testing of SparkRx; and (8) any other condition, comorbidity, or event that may prevent the potential participant from adhering to the protocol or benefitting from the intervention (eg, coercion or low level of cognitive functioning) or will prevent investigators from being able to ensure safety.

### Recruitment, Consent, and Screening

Recruitment is conducted through direct advertisement, including web-based and print media advertisements, flyers, word of mouth, and health care provider referrals. Potential participants (and legal guardians, if applicable) are directed to a study website where they can evaluate their preliminary eligibility to participate and sign up for a videoconferencing-based consent and onboarding session if they are preliminarily eligible to participate. During the session, potential participants and legal guardians (if required) provide informed consent or assent. The consent form provides information about the potential uses and disclosures of participant data, as well as the security practices for maintaining data. Specifically, the consent form provides a description of the parties who may gain access to participant data (eg, Data and Safety Monitoring Board, US Food and Drug Administration, and third-party vendors required for the operation of the study app and web platform). Third-party vendors are required by contract to maintain the confidentiality of participant data and to follow applicable data protection laws. Participants are informed that study staff will protect information from disclosure to others to the extent required by law but are obliged to share information under certain circumstances, such as in cases of suspected abuse, or indications of imminent harm to self or others. The consent form also discusses the risk of a loss of confidentiality and describes a few of the security features in place to reduce the likelihood of such a breach, including firewalls and end-to-end data encryption. Following consenting procedures, participants complete a screening/baseline PHQ-8 assessment and are screened for all remaining eligibility criteria. For potential participants younger than 18 years, self-report confirmation of eligibility is conducted privately with the minors and again with their legal guardians to increase the likelihood that sensitive information relevant to determining ineligibility (eg, active suicidal ideation with intent) is disclosed. Research coordinators confirm the licensure of participants’ health care providers in the National Provider Identifier registry and inform providers of their patients’ participation in the study via phone message. Eligible participants are enrolled into the study, complete baseline assessments, and receive instructions for downloading their assigned study app onto their mobile device. Participants are also provided with a safety plan template [50] and instructions for use as a personal resource.

### Randomization With Stratification

A permuted block randomization schedule is used to randomly assign participants in a 1:1 ratio to either the treatment arm or the control arm. Stratification factors for randomization include baseline total PHQ-8 score (<15 or ≥15) and current antidepressant medication use (yes or no).

### Intervention Arms

#### Overview

Participants are randomized to receive either the treatment app (SparkRx) or a control app (eUC). Both apps deliver weekly questionnaires for participants to complete. User guides for each app include instructions on app usage, study team contact information, crisis resources, and troubleshooting help.

#### Treatment Arm: SparkRx (Investigational)

SparkRx is a 5-week, self-guided multilevel program based on clinically validated behavioral activation protocols [44,45] to treat adolescent symptoms of depression (see Multimedia Appendix 1). In the first level, participants learn about the deleterious cycle of negative mood triggers and behavioral avoidance patterns in depression and practice identifying their negative mood-behavior patterns by tracking their mood in the app. In the second level, participants learn to distinguish between “Up” and “Down” activities and value-based activity scheduling is introduced. In the third level, participants practice scheduling activities to perform outside of the app. Push notifications remind and encourage participants to log their moods, complete their scheduled activities, and review each activity and their mood after scheduled activity times have elapsed. Participants engage in guided problem-solving to overcome challenges in the completion of their scheduled activities. In levels 4 and 5, participants learn mindfulness techniques to combat avoidance patterns and develop an action plan to maintain gains after SparkRx program completion. Levels and tasks within each level progress in a linear fashion (ie, each task or level must be completed to progress to the next task or level). Participants are encouraged to complete 1 level per week, though they are at liberty to complete the levels at their own pace without penalty. Certain on-demand resources can be accessed in the app at any time, including crisis resources.

#### Control Arm: eUC

Usual care is broadly defined as any of the existing treatment options for symptoms of depression, including active monitoring of depressive symptoms and suicidality, supportive counseling by a health care provider, psychosocial support interventions, collaborative care (eg, facilitation of parental and patient self-management, referral for peer support or other community, or school-based behavioral health programs), psychoeducation, complementary and alternative medicine approaches, psychotherapy (eg, behavioral treatment, interpersonal therapy, or CBT), pharmacotherapy for mood problems, visit to a primary care provider, behavioral or mental health specialist or therapist, counselor or coach for a mood disorder. It also includes the option of obtaining no treatment for symptoms of depression. For purposes of this study, usual care is considered enhanced (eUC) by prompting participants to complete a weekly PHQ-8 assessment and side effect screen (Participant Symptom Check) in the control mobile app, providing a safety plan template, and clinician-based safety monitoring (with corresponding clinical reach out, as needed).

### Blinding (Investigator)

Study staff and investigators are not directly involved in the collection of any efficacy outcome data. These data are collected directly from participants through the study app and a secure web portal. The proprietary electronic data collection system used in this trial (Limbix Synapse) enables selective blinding or unblinding of designated individuals; thus, study staff who enroll participants, monitor assessment completion and send reminders to complete assessments, and directly correspond with participants about technical difficulties are not exposed to any data fields that contain implicitly or explicitly unblinding information.

Randomization is performed automatically upon entry of relevant data fields into Synapse during enrollment, and the deployment of assigned interventions takes place without the need for study staff involvement and without exposure of treatment assignments to study staff. Specifically, all participants download a single study app onto their mobile devices. Content specific to a participant’s assigned treatment arm is displayed only after they log in to the app for the first time and confirm that study staff are unable to see their mobile device screen. Participants are asked not to reveal their assigned treatment arm in communications with study staff.

Study coordinators and clinicians involved in safety monitoring are unblinded to treatment assignment by necessity, as monitoring includes a review of freeform text entered into only the SparkRx app, clinical reach out in response to identified safety concerns, and the identification and categorization of adverse device effects (see the *Safety Monitoring* section for details). Unblinded study staff are instructed not to discuss treatment allocation or any potentially unblinding information with blinded study team members.

### Assessments

#### Overview

Assessments take place at baseline, weekly during the intervention period, at the postintervention time point, and at 1-month follow-up (see Table 1 for the schedule of assessments). Baseline assessments are completed during the consent and onboarding session prior to mobile app download. Participants complete weekly assessments during the intervention period in their assigned mobile app. In-app push notifications and text messages are sent to remind participants to complete these assessments within a 4-day response window. Participants in the treatment arm are unable to continue engaging with content in the app until they have completed (or actively skipped) the weekly assessment or until their response window for a given week has closed. Legal guardians (if applicable) complete weekly proxy assessments in a secure web portal and receive reminders to do so. At the postintervention time point (immediately following the intervention period) and again at 1-month follow-up, participants (and legal guardians, if applicable) complete assessments in a secure web portal. The window for completion of postintervention and 1-month follow-up assessments is 2 weeks. Participants who do not complete 1-month follow-up assessments within the 2-week window are considered lost to follow-up. No collateral reports are obtained for participants older than 18 years.

**Table 1:** Schedule of assessments.

	Eligibility screening	Baseline	Weekly during the intervention period	Postintervention	1-month follow-up
Medication use screener	✓				
Patient Health Questionnaire^a,b^		✓	✓	✓	✓
Demographics and medical history questionnaire^a^		✓			
Behavioral Activation for Depression Scale–Short Form		✓		✓	✓
Generalized Anxiety Disorder–7		✓		✓	✓
Pediatric Quality of Life Enjoyment and Satisfaction Questionnaire^a^		✓		✓	✓
Absenteeism questionnaire^a^		✓		✓	✓
Health care utilization questionnaire^a^		✓		✓	✓
Participant symptom check^a^			✓	✓	✓
Systems Usability Scale				✓	
User Engagement Scale–Short Form				✓	

^a^A legal guardian proxy is additionally administered when participants are younger than 18 years.

^b^A single Patient Health Questionnaire–8 is administered to participants at the consent and onboarding session for purposes of both eligibility screening and baseline assessment.

#### Primary Outcome

The primary outcome is depressive symptom severity as measured by the PHQ-8, an 8-item participant-reported measure used to screen for depression and to establish depression severity [48]. The PHQ-8 was selected as the primary end point to align closely with a tool used for initial screening (and severity assessment) of depressive symptoms in clinical practice. Whereas the 9-item Patient Health Questionnaire is often used in clinical settings, the PHQ-8 (which drops an item related to suicidality) is commonly used in research contexts that are not geared toward clinically responding to reports of suicidality [48].

The total score ranges from 0 to 24, with a higher score indicating greater depression symptom severity. Scores of 5, 10, 15, and 20 represent thresholds for mild, moderate, moderately severe, and severe symptom severity, respectively; here, we define the minimal clinically important difference to be a 5-point reduction in total score from baseline [51,52].

#### Secondary Outcomes

The total PHQ-8 score will also be used to compute the trial’s secondary outcomes, which include the (1) proportion of participants meeting criteria for intervention response, defined as a 50% reduction in symptoms from baseline to postintervention [47,53-56]; (2) proportion of participants meeting criteria for remission, defined as a score less than 5 at the postintervention time point [53,57]; (3) depressive symptom severity at 1-month follow-up; and (4) proportion of participants meeting criteria for a clinically meaningful reduction in symptom severity, defined as a 5-point or greater reduction in PHQ-8 score from baseline to the postintervention time point.

#### Tertiary and Exploratory Outcomes

As described below, a series of additional participant- and legal guardian–reported assessments are collected to investigate the potential effects of the SparkRx digital therapeutic on other clinical and functional domains, as well as its perceived usability and degree of engagement.

Anxiety symptoms are assessed with the Generalized Anxiety Disorder–7 (GAD-7) scale. The GAD-7 is a validated 7-item participant-reported assessment for generalized anxiety disorder [58]. The total score ranges from 0 to 21, with a higher score indicating greater anxiety symptom severity.

Quality of life will be assessed with the Pediatric Quality of Life Enjoyment and Satisfaction Questionnaire (PQ-LES-Q), a 15-item participant-reported self-administered questionnaire that captures life satisfaction over the past week [59]. Each question is rated on a 5-point scale from 1 (very poor) to 5 (very good). The first 14 items are summed to form a total score, with higher scores indicating higher quality of life. The 15th item (“Overall, how has your life been?”) is a stand-alone item. For participants younger than 18 years, a legal guardian proxy version of the PQ-LES-Q is administered to legal guardians.

Depression-associated behavior is assessed with the Behavioral Activation for Depression Scale-Short Form (BADS-SF). The BADS-SF [60] consists of 9 participant-reported questions assessing behavioral activation and avoidance over the previous week, each rated on a 7-point scale ranging from 0 (not at all) to 6 (completely). This form can be used to track changes in behaviors that are targeted by behavioral activation therapy. It examines changes in areas such as activation, avoidance or rumination, work or school impairment, and social impairment. The total score ranges from 0 to 54, with higher scores indicating higher activation.

The usability of the mobile app is assessed with the participant-reported System Usability Scale (SUS) [61]. The SUS consists of a 10-item questionnaire with 5 response options for respondents, ranging from strongly agree to strongly disagree. User engagement is assessed with the User Engagement Scale–Short Form [62], a participant-reported, statistically reliable measure that is tailored to the specific device being tested (ie, the study app). The form has 12 items and uses a 5-point Likert scale to evaluate domains such as aesthetic appeal, focused attention, perceived usability, and reward. Each subscale score ranges from 1 to 5, with higher scores indicating higher levels of each scale. The total score also ranges from 1 to 5, with higher scores indicating more engagement.

Absenteeism is assessed with study-specific adaptations of the absenteeism and presenteeism questions of the World Health Organization’s Health and Work Performance Questionnaire [63] that solicit information from participants (and legal guardians, if applicable) about missed time at work or school attributable to the participant’s mental health condition over the past 4 weeks.

Finally, the use of health care resources is evaluated with a brief questionnaire assessing the use of health care resources such as hospital visits and medical appointments over the past 4 weeks.

#### Treatment Compliance Outcomes

Treatment compliance for those assigned to use SparkRx is assessed descriptively by examining the rates of module and program completion, as well as the amount of time spent in the app over the course of 5 weeks and the number of participants who engaged with the app on each day of the intervention period.

### Safety Monitoring and Safety Outcomes

#### Safety Monitoring

Safety is monitored during consent and onboarding sessions and throughout study participation. In this trial, clinical concerns are defined as any negative experiences or symptoms reported by a participant or a legal guardian, whether or not they are thought to be associated with participation in the study. Examples of clinical concerns include past or current suicidal ideation, self-harm, worsening of depression symptoms, new symptoms, changes in medication or treatment status, or hospitalizations. Sources for potential clinical concerns include (1) freeform text input into the SparkRx app or study questionnaires; (2) communication from participants or legal guardians to study staff during the consent and onboarding session or study participation; (3) patterns of responses to PHQ-8 assessments, as defined below; and (4) responses to the Participant Symptom Check that is deployed to participants (and legal guardians, if appropriate) weekly during the intervention period, at the postintervention time point, and at 1-month follow-up. Responses to PHQ-8 assessments are considered to be clinical concerns if they suggest (1) temporary clinical deterioration, defined as a PHQ-8 score >15 that reflects a 5-point or more increase from baseline, or (2) sustained elevated symptom severity, defined as a total PHQ-8 score greater than 20 for 2 or more consecutive weeks. In addition, a built-in algorithm in the SparkRx app compares all freeform text input into the app to a defined list of risk words and phrases. When a match occurs, the participant receives a pop-up alert directing them to the crisis resources page; study staff are automatically notified about potential clinical concerns involving the use of risk words or phrases. All identified clinical concerns are logged by a study staff member and reviewed by a study clinician daily. If clinical involvement for a logged clinical concern is indicated for the purpose of clinical safety management or to investigate the presence of a possible adverse event (AE), the study clinician contacts the participant and legal guardian (if applicable) to further evaluate clinical status and make determinations regarding continued participation in the study.

#### AE Classification

AEs, per the US Food and Drug Administration’s guidance [64], are identified from logged clinical concerns during regular reviews by the study clinician. Each AE is then preliminarily categorized by expectedness, seriousness, and the likelihood of relatedness to the SparkRx app (“not possible,” “possible,” “probable,” or “definite”), as well as whether it constitutes a psychiatric or nonpsychiatric occurrence.

Given the trial population, the following AEs are classified as “expected”: (1) passive or active suicidal ideation, with or without intent to act; (2) clinically significant deterioration in depressive symptoms, including temporary clinical deterioration as defined in the *Safety Monitoring* section; (3) nonsuicidal self-injury ideation; and (4) nonsuicidal self-injury not resulting in the need for medical care or intervention. All other types of AEs are classified as “unexpected.”

Definitions of AE seriousness and adverse device effects follow the guidance provided by the International Organization for Standardization 14155:2020. “Psychiatric” AEs constitute experiences that are related to the presence of a mental disorder, symptom of a disorder, or issue, as defined in the *Diagnostic and Statistical Manual of Mental Disorders, Fifth Edition* (DSM-5) [65].

Following preliminary categorization by a study clinician, a second independent clinician, not otherwise associated with the study, completes the final categorization of all AEs.

### Statistical Analysis

#### Overview

Efficacy and safety analyses will be conducted by an independent statistician external to the sponsor organization who is otherwise not involved in the design or conduct of the trial. A statistical analysis plan, finalized prior to the conclusion of data collection, will be followed that includes detailed provisions for the handling of missing data. The plan will be made public as part of results reporting on ClinicalTrials.gov (NCT05462652).

#### Populations

The primary population for all analyses is defined according to the intention-to-treat (ITT) principle, whereby all participants will be included according to their assigned study arm at baseline, regardless of adherence to study protocol.

Secondary analyses of the primary and secondary outcomes will be conducted with the following additional populations: (1) a modified ITT population will exclude from analysis participants who were found to not meet eligibility criteria after randomization and individuals without any postrandomization data. This population is included to ensure that effects are observed with the appropriate intended sample. In addition, this population allows for the handling of missing data without the need to impute values based solely on baseline data. (2) A per-protocol population will include individuals who complete, at minimum, the postintervention PHQ-8 assessment and, for participants in the SparkRx arm, meet a minimum engagement threshold of completing 2 app modules. This population is intended to estimate treatment effects in participants who adhere to the study and treatment protocol. (3) A moderate-severe population will include participants from the ITT population whose baseline PHQ-8 score was 10 or greater. This population is intended to ensure that efficacy outcomes are not masked by floor effects.

#### Analysis of Primary and Secondary Outcomes

A mixed effect model with an appropriate correlation matrix will be implemented to evaluate the effect of treatment on PHQ-8 scores at the postintervention time point. Time point, treatment arm, time point by treatment interaction, baseline PHQ-8 score, and antidepressant medication status will be included in the model as fixed effects. For analysis of the ITT population, missing PHQ-8 total scores will be imputed with multiple imputation within treatment arm under the assumption that data are missing at random; 20 imputed data sets will be analyzed independently and then combined [66]. As a sensitivity analysis, this model will be repeated without imputation using the modified ITT population, which includes only participants with at least 1 postbaseline data point.

For descriptive purposes, the mean change from baseline (and 95% CIs) in the SparkRx arm will be estimated to determine whether the change in this group meets or exceeds criteria for minimal clinically important difference (a 5-point improvement) and whether a 5-point change was contained or surpassed by 95% CI.

To analyze the secondary outcomes, we will compute the number and proportion of participants meeting the criteria for intervention response, remission, and clinically meaningful reduction in severity at postintervention and compare the rates between treatment arms using chi-square tests. If counts are low, Fisher exact tests will be used. As above, the 20 imputed data sets will be analyzed independently and combined. All tests will be 2-tailed.

#### Control of Type I Error in Primary and Secondary Outcome Analyses

The primary outcome analysis, conducted with the ITT population, will be tested with a 2-tailed α set at .05. To control for multiple comparisons, a serial gatekeeping approach will be applied, whereby the secondary outcomes will be tested only upon obtaining a significant primary outcome test and in the order in which they are listed in the Secondary Outcome section (see above). All subsequent outcomes will be considered to be nonsignificant following the first *P* value greater than α=.05.

#### Additional Planned Analyses of the Primary and Secondary Outcomes

Analyses will be repeated within the 4 subgroups: aged <18 years at enrollment, aged ≥18 years at enrollment, baseline PHQ-8 score <15, and baseline PHQ-8 score ≥15. *P* values will be reported; however, because the protocol was not powered for comparisons within such subgroups, they should be considered purely descriptive.

#### Analyses of Tertiary and Exploratory Outcomes

Appropriate models (eg, analyses of covariance) will be used to compare continuous clinical outcomes (eg, GAD-7, BADS-SF, PQ-LES-Q, and legal guardian PHQ-8 proxy) at the postintervention time point between treatment arms. Baseline scores will be included in models as appropriate. Changes in health care utilization and absenteeism from baseline to postintervention will be examined descriptively.

Moderation analyses will test whether demographic and other participant features (eg, baseline PHQ-8 score, antidepressant medication use, sex or gender, and age) influence the effects of treatment on PHQ-8 outcomes. Mediation analyses will be used to determine whether the degree to which participants engaged with SparkRx (eg, module completion rate) explains its effect on PHQ-8 scores. The usability and engagement of the SparkRx app (SUS and User Engagement Scale–Short Form) will be examined descriptively.

#### Analysis of Safety Outcomes

The rate of psychiatric SAEs (irrespective of device relatedness) derived from the Participant Symptom Check (side effect screen) and PHQ-8–based deteriorations will be compared between SparkRx and eUC groups using the Fisher exact test. For purposes of this comparison, psychiatric SAEs reported via app freeform text will be excluded, as freeform text is unique to the SparkRx arm.

### Control of Bias

As Limbix Health Inc is the sponsor-investigator of this trial, there is a risk of bias due to study staff’s conflicts of interest. This risk is mitigated in a number of ways. First, the efficacy end points are participant reported and digitally input by the participant directly into a password-protected, web-based assessment portal or into their study app. After the baseline session, data are not collected in the presence of study staff members. Thus, study staff members will not be directly involved in the capture of efficacy data. Except in cases of clinical reach out to ensure participant safety, all communication with study participants will be conducted by study staff blinded to treatment assignments to prevent bias. Second, to reduce bias related to the collection of safety outcomes, an independent clinician with no other role in the study nor conflict of interest will review all AEs and provide final classifications of AE seriousness and device relatedness for use in data analysis. Third, an independent statistician will conduct statistical analyses for the study based on a Statistical Analysis Plan to be finalized prior to data transfer.

## Results

As of May 2023, enrollment into the study has concluded; 223 were participants randomized. The analysis of the efficacy and safety data is expected to be completed by Fall 2023.

## Discussion

### Expected Findings

While SparkRx is based on well-validated therapeutic behavioral activation protocols for depression, this RCT represents one of the first assessments of their efficacy in treating symptoms of depression as deployed to adolescents via a self-guided mobile app. For our primary outcome, we hypothesize that participants assigned to the SparkRx arm will report lower total PHQ-8 scores at postintervention compared to those in the eUC arm. For our secondary outcomes, we hypothesize that there will be higher rates of intervention response, remission, and clinically meaningful reduction in severity in the SparkRx arm than in the eUC arm. In addition, we expect to observe a durable effect of SparkRx as indicated by significantly lower PHQ-8 scores at 1-month follow-up in participants who received the SparkRx app compared to those who did not. Similarly, we hypothesize that participants in the treatment arm will report experiencing fewer symptoms of anxiety, greater quality of life, and higher levels of behavioral activation (lower levels of behavioral avoidance) at the postintervention time point than participants in the control arm. Finally, we anticipate that there will be statistically no more psychiatric SAEs in the SparkRx arm than in the eUC arm, supporting the overall safety of the SparkRx intervention.

### Potential Problems

Poor adherence and low levels of engagement are common challenges with self-guided digital therapies [67]. As such, user engagement was a primary goal in the development of the SparkRx intervention [68]; the app was co-designed with a teen advisory council and iterated through rounds of user research and diary studies among adolescents with and without depression symptoms. For this trial, a few additional strategies are being implemented to encourage adherence to the study protocol. In addition to SparkRx push notifications that encourage engagement with the app and scheduled activities, both automatic and manual text messages are sent to remind participants to complete assessments. Adherence is also incentivized by compensation that is tied to discrete study components (attending the onboarding session and completing each set of questionnaires).

### Dissemination

The primary results of this trial will be submitted to a peer-reviewed journal, irrespective of magnitude or direction of effect, and findings will be presented at scientific conferences. A lay report of findings will also be prepared for dissemination to trial participants (upon request) and to broader audiences.

### Conclusions

During this mental health crisis, adolescents are in urgent need of accessible and quality care. Digital therapeutics, such as SparkRx, can bring such care to those who might otherwise have to wait months to see a provider or who face barriers to receiving care, such as high costs or stigma against traditional mental health treatment. To our knowledge, this is the first digital therapeutic offering CBT-based content specifically designed for adolescents with symptoms of depression, and this RCT is the first fully powered test of the efficacy of SparkRx. Support for our hypotheses would increase not only the scientific evidence base for SparkRx specifically but also more broadly for the model of using self-guided, adjunct digital therapeutics in the treatment of mental health disorders in adolescents.

## Appendix

Multimedia Appendix 1 Screenshots depicting aspects of the SparkRx app (including psychoeducational programming, mood logging, and value-based activity scheduling and tracking).

## Abbreviations

AE: adverse event
BADS-SF: Behavioral Activation for Depression Scale–Short Form
CBT: cognitive behavioral therapy
DSM-5: Diagnostic and Statistical Manual of Mental Disorders, Fifth Edition
eUC: assessment-enhanced usual care
GAD-7: Generalized Anxiety Disorder–7
HIPAA: Health Insurance Portability and Accountability Act
ITT: intention-to-treat
PHQ-8: 8-item Patient Health Questionnaire
PQ-LES-Q: Pediatric Quality of Life Enjoyment and Satisfaction Questionnaire
RCT: randomized controlled trial
SAE: serious adverse event
SUS: System Usability Scale
