# Supplementary figures and images for: Assessing the Efficacy and Safety of a Digital Therapeutic for Symptoms of Depression in Adolescents: Protocol for a Randomized Controlled Trial
Source: JMIR Res Protoc. 2023 Nov 16;12:e48740. doi: 10.2196/48740 (PMC10690536; doi:10.2196/48740)

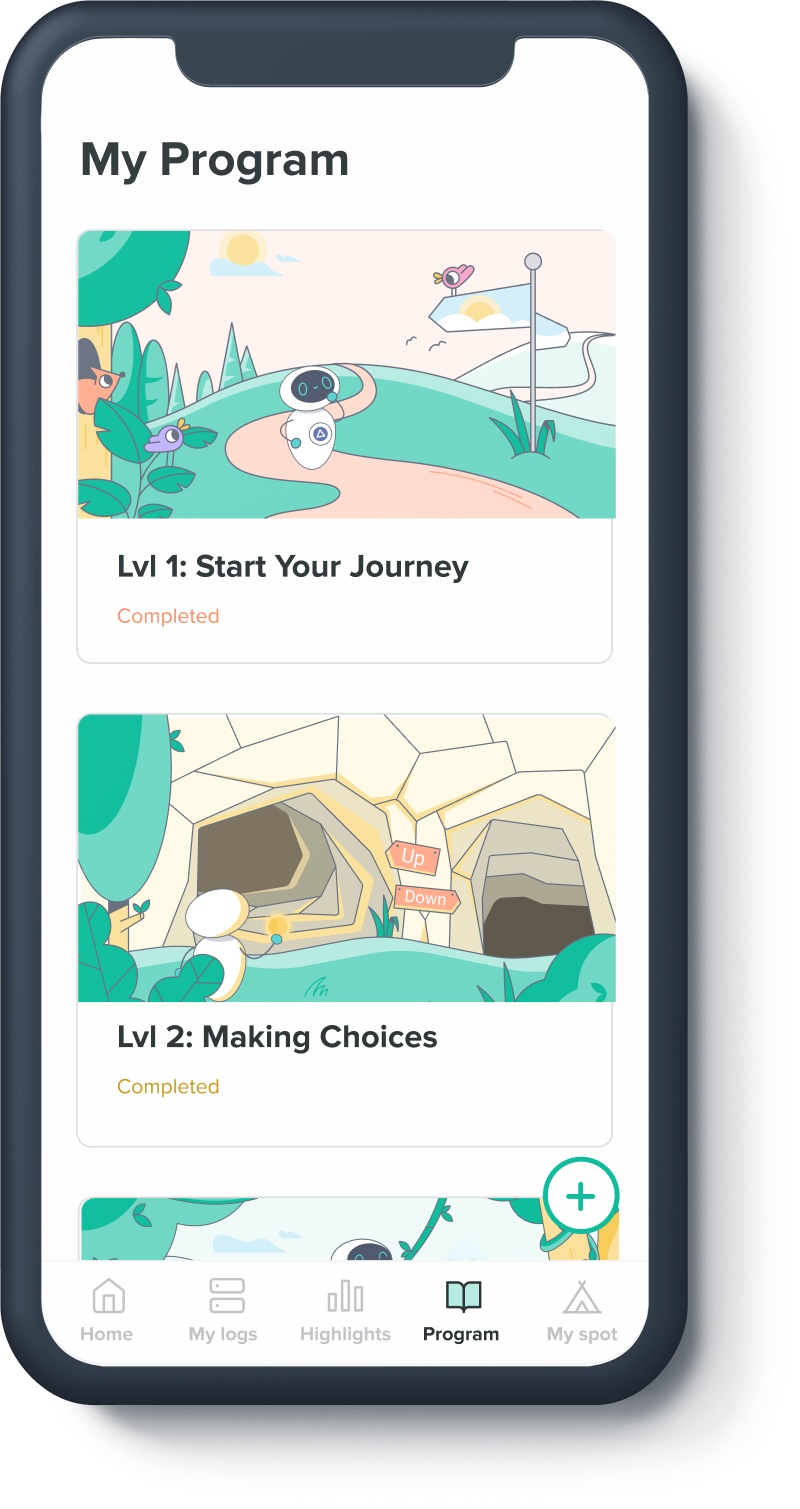

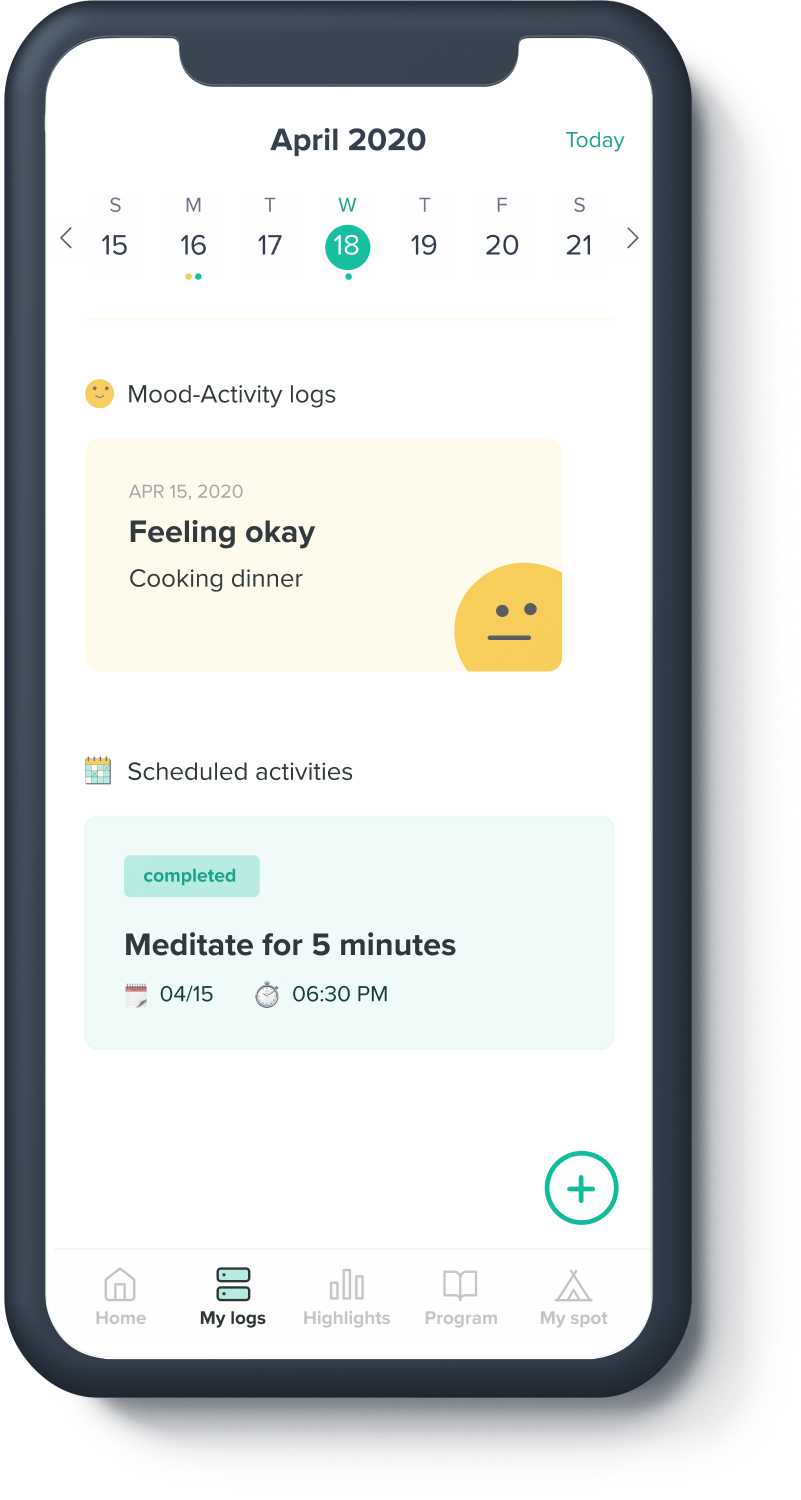

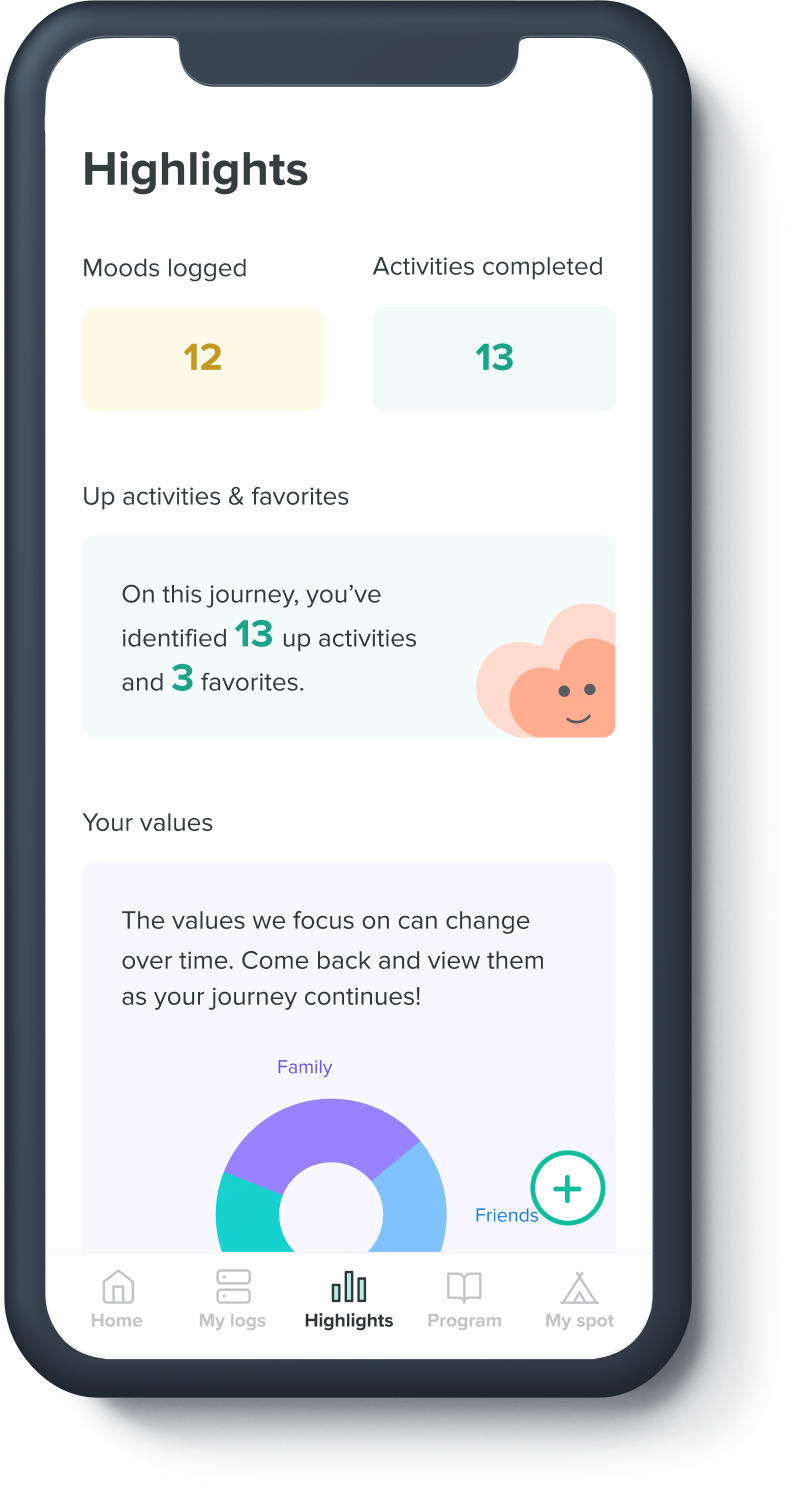

Supplement: Multimedia Appendix 1 [file resprot_v12i1e48740_app1.docx]
